# Relationship between pre-extubation positive endexpiratory pressure and oxygenation after coronary artery bypass grafting

**DOI:** 10.5935/1678-9741.20150063

**Published:** 2015

**Authors:** Marcos Aurélio Barboza de Oliveira, Antonio Carlos Brandi, Carlos Alberto dos Santos, Paulo Henrique Husseini Botelho

**Affiliations:** Faculdade de Medicina de São José do Rio Preto, São José do Rio Preto, São Paulo, Brazil; UNIFEV, Votuporanga, SP, Brazil;; Hospital de Base São José do Rio Preto, São José do Rio Preto, SP, Brazil.

At least one methodological error can be found in most randomized clinical trials that have been published in scientific journals; that is, some authors make such error compromising the reliability of the entire study. Some studies not even show the data in a systematic way; in consequence, even the best reviewers are not able to certify the results^[1]^.

To provide a more reliable and easily certified content by the reviewers, a selected group of researchers, methodologists, statisticians and scientific journal editors have met to create a set of guidelines in a Checklist format^[2,3]^.

"Consolidated Standards for Reporting Trials" was the generic name given for such guidelines or CONSORT Statement as it is known. In 1996^[4]^, the first version was published, under some changes until 2010^[2]^; remaining this as the current version.

This set of rules was initially designed to guide randomized clinical trials; being inappropriate for surgical work, in which there is greater difficulty in applying the blind condition for patients and evaluators to minimize either variations in surgical techniques as differences on the surgeons' experience who perform these procedures^[5]^. For this purpose, it was published an extension of the CONSORT Statement in 2008 that provided specific recommendations for reporting randomized trials for non-pharmacological treatment (CONSORT-NPT)^[6,7]^.

For these non-pharmacological work, there are specific instructions for each section of the paper; such as the Title with the word "randomized", Abstract with the blind condition of the study, Method calculating the minimum number of patients to be included in each group, flow-chart, among others. A full description of such rules can be found in Boutron et al.^[6]^work.

The work entitled "Relationship between pre-extubation positive end-expiratory pressure and oxygenation after coronary artery bypass grafting" of Borges et al.^[8]^published in this issue of the Brazilian Journal of Cardiovascular Surgery includes some of these criteria. At first, the authors define the study as randomized in the Abstract; the Methodology shows clear inclusion and exclusion criteria presented in text format, detailed description of the Methods to be applied to patients according to their groups, detailed statistics and, in the Results, graphical flow-chart type of the number of patients in each study phase.

We congratulate the authors for the effort of reporting such results obeying rules that help ensure a high standard of reliability in their study. Nevertheless, we should draw attention that there are several items of CONSORT Statement that have been omitted, such as calculating the number of patients in each group; the kind of randomization method that was chosen; the critical analysis of the external validity of the work as well as the discussion of the study limitations. These observations are consistent with Hopewell et al.^[9]^ findings since they once have reported the conclusion in scientific magazines, CONSORT Statement is included for instructions to authors, there are more items of the Checklist in each clinical trial than in those in which this method is not mentioned.

Randomized clinical trials are reliable sources of scientific information; however, the use of inappropriate methodology can lead to false conclusions, undermining the reliability of the study. Thus, the implementation of this methodology helps editors to assess trial quality and assures readers the reliability of conclusions reported at the end of each paper. It is our role, as reviewers and editors of the Brazilian Journal of Cardiovascular Surgery, to encourage authors to use the CONSORT Statement not only to facilitate the certification process of the study's conclusions, but also in order to increase the quality of their own manuscripts.

**Marcos Aurélio Barboza de Oliveira, PhD - Faculdade de Medicina de São José do
Rio Preto, São José do Rio Preto, São Paulo, Brazil; UNIFEV, Votuporanga, SP,
Brazil;** **Antonio Carlos Brandi, PhD; Carlos Alberto dos Santos, PhD; Paulo Henrique
Husseini Botelho, MD - Hospital de Base São José do Rio Preto, São José do Rio
Preto, SP, Brazil.**
